# Quantifying respiratory complications post-adenotonsillectomy in patients with normal or inconclusive overnight oximetry

**DOI:** 10.1186/1916-0216-42-50

**Published:** 2013-10-09

**Authors:** Terence TN Lee, Claire E Lefebvre, Nathalie E Gans, Sam J Daniel

**Affiliations:** 1 Department of Otolaryngology, Head and Neck Surgery, Montreal Children’s Hospital, McGill University, Montreal, Quebec, Canada

**Keywords:** Adenotonsillectomy, Children, Polysomnography, Sleep-disordered breathing

## Abstract

**Background:**

Children with sleep-disordered breathing (SDB) are at risk of developing post-operative respiratory complications following adenotonsillectomy (T&A). Our goal was to describe and quantify these complications following T&A in children with clinical SDB but with a pre-operative overnight home oximetry score of “normal/inconclusive” (McGill Oximetry Score (MOS) of 1), and to determine whether these children could safely undergo surgery in peripheral hospitals or outpatient surgical centers.

**Methods:**

We performed a retrospective chart review of patients 3 years and older who had T&A between 2003 and 2010 at 2 of our institution’s hospitals. To be included in the study, in addition to not having severe comorbidities, children had to have undergone an overnight home oximetry within 12 months of surgery that was normal or inconclusive (MOS of 1). This was defined as fewer than 3 episodes of oxygen desaturation below 90% and stable baseline saturation over 95%. Medical charts were reviewed for major and minor postoperative respiratory complications. The main outcome measure was post-T&A respiratory complications.

**Results:**

Out of 2708 T&A patients, 231 met the inclusion criteria. No patient had a major postoperative respiratory complication requiring re-intubation or admission to the intensive care unit. Five patients (2.16%) had minor respiratory complications but only one required admission to the ward.

**Conclusions:**

An overnight home oximetry that is “normal/inconclusive” (MOS of 1) can be used as a screening tool to identify patients with sleep-disordered breathing who can be safely sent to peripheral hospitals or outpatient surgical centers for T&A.

## Background

Sleep-disordered breathing (SDB), characterized by any abnormality in nighttime respiratory pattern or ventilation, affects 6-12% of children [1-4], and is the leading indication for adenotonsillectomy (T&A) in the United States [5,6]. Even if SDB does not meet the criteria for obstructive sleep apnea (OSA) as defined by a positive polysomnography (PSG), it needs to be promptly identified and treated, as there is evidence that it may have negative long-term consequences on cognitive development [4,7-13]. In Canada, the T&A rate is as high as 19 per 10 000 children or adolescents [14], and offloading these surgeries from tertiary care hospital centers to peripheral hospitals or outpatient surgical centers where they can be performed more quickly would have an important positive impact on healthcare resource utilization. However, because these peripheral or outpatient centers are not equipped to provide emergency resuscitative support after hours, only children at a low risk of major post-operative complications should be referred there.

At our center, overnight oximetry is used to assess severity of suspected SDB to facilitate prioritization for T&A. Abnormal oximetries, in particular, have been shown to be predictive of post-T&A respiratory complications [15-17]. Our center uses a 4-level severity system, the McGill Oximety Score (MOS), that was developed and retrospectively validated by Nixon et al. on the basis of the depth and number of desaturation occurrences [18]. A positive predictive value of abnormal (MOS of 2, 3 or 4) and normal/inconclusive (MOS of 1) is 10% and 3%, respectively, for major respiratory complications [18]. However, in those with a MOS of 1, the authors did not specifically evaluate whether the surgeries can be safely performed at a peripheral or outpatient center. They included patients who were less than 3 years old, who, according to current clinical practice guidelines [19], should be admitted post-operatively. In addition, their definition of major respiratory intervention was broad, including maneuvers that can be performed at an outpatient center, such as placement of an oropharyngeal/nasopharyngeal airway and ventilation with a bag and mask.

We hypothesize that a MOS 1 can accurately identify children with SDB who are at low risk of developing serious post-operative respiratory complications, such as admission to pediatric intensive care unit (ICU) or reintubation. It could therefore be used as a screening tool to determine which children are suitable to undergo T&A in peripheral hospitals or outpatient surgical centers, shortening surgical wait times in tertiary centers where high-risk patients need to be followed. In this retrospective study, our objective was to describe and quantify post-operative respiratory complications of children aged 3 and older with MOS of 1 undergoing T&A.

## Methods

### Study design and population

This retrospective chart review was approved by the McGill University Health Centre’s Montreal Children’s Hospital (Montreal, Quebec, Canada) ethics committee. Medical archivists at the MCH retrieved charts from all patients who underwent T&A or tonsillectomy using Canadian Classification of Health procedure codes 1.FR.89.WJ and 1.FR.89.LA respectively. All patients whose charts contained a pre-operative home oximetry that was “normal/inconclusive” (MOS 1) were then included and their entire chart reviewed. The MOS of 1 (“normal/inconclusive”) is defined as fewer than 3 episodes of oxygen desaturation below 90%, no desaturations below 85%, and stable baseline saturation over 95% [18].

T&A’s were performed between January 1, 2003 and December 5, 2009 at the Montreal Children’s Hospital and from April to December 2010 at Montreal’s Verdun Hospital. At the time of data collection, T&A’s performed at the MCH after December 2009 had not yet been archived and were therefore unavailable for chart review. To expand our study pool, we included patients from Verdun Hospital whose surgeries are performed by MCH surgeons since 2010. Children operated at both centers are from the same referral pool. Patients sent to Verdun Hospital must have had a MOS of 1 performed within a year prior to surgery and must not have neurological, cardiac or respiratory abnormalities as well as any genetic syndromes.

The children were included if “hypertrophic tonsils”, “T&A hypertrophy”, “rule out lymphoma” or “OSA” were the indications for surgery as these were felt to reflect the presence of large tonsils possibly contributing to airway obstruction. Surgeries performed for other indications such as “recurrent tonsillitis” or “tonsillar abscess” were omitted. For consistency, the final pre-operative diagnosis as indicated by the treating surgeon was chosen and superseded any discrepant diagnoses in the chart.

In our study, patients were excluded if they had undergone the home oximetry more than 12 months prior to surgery. In cases where more than one overnight oximetry had been performed prior to surgery, the one dated closest to the surgery was chosen. For consistency of equipment and monitoring environment, overnight oximetries performed privately or on an in-patient basis were excluded. When, at the physician’s discretion, PSG was performed following the “normal/inconclusive” (MOS 1) home oximetry and showed moderate to severe OSA (i.e. when the oximetry result was determined to be falsely negative), the child was excluded from the study.

**Figure 1:**
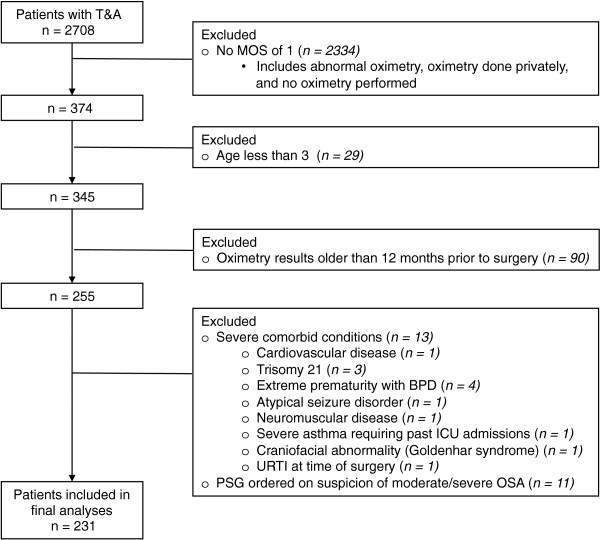
Flow chart for 2708 patients with T&A and the 231 patients included in the study.

Patients less than 3 years of age were excluded from the study in keeping with current safety guidelines that recommend post-operative admission for these patients [19]. In addition, we excluded 13 patients with severe comorbidities (Figure 1): Cardiovascular disease (1), Trisomy 21 (3), extreme prematurity with BPD (4), atypical seizure disorder (1), neuromuscular disease (1), severe asthma requiring past ICU admissions (1), craniofacial abnormality (Goldenhar syndrome) (1), and URTI at time of surgery (1).

### Variables collected

Pertinent history, operative and postoperative information were gathered from the patients’ paper charts. Risk factors were recorded if they were thought to contribute to post-operative respiratory complications. These were gender, age, weight percentile for age, preoperative medical status, time lapse from oximetry to surgery, surgeon and duration of surgery. Post-operative events of interest such as respiratory complications and medical interventions were identified.

### Outcome and definitions

The main outcome of interest was major post-operative respiratory complications requiring intubation or admission to ICU. Secondary outcome was minor post-operative respiratory complications. These were defined as any maneuver performed in an attempt to correct or monitor witnessed respiratory difficulty or oxygen desaturation. Interventions included: jaw thrust maneuver, insertion of oral or nasal airway, administration of supplemental oxygen or cold humidity, bagging, CPAP or admission to the ward for observation.

We defined the postoperative period from the moment of admission to the recovery room until discharge from hospital. Standard protocol is for children to be extubated in the operating room and transferred to the Post-Anesthesia care unit (PACU) where they receive an oxygen mask close to their face for 15–30 minutes. Oxygen saturation is monitored with a Nellcor N200 pulse oximeter until 30 minutes after opioid administration. A discharge criterion from the recovery room is documentation of room air saturation greater than 95% while awake.

### Analysis

Descriptive statistics were used to compare baseline characteristics for complications and admissions (PASW Statistics Version 18.0). Continuous variables are presented as mean ± SD or median and were compared by Student’s t test or Mann–Whitney U test. Dichotomous variables are presented as percentage and were compared by chi-square test.

## Results

Between January 1, 2003 and December 5, 2009 and from April to December 2010, 374 out of 2708 T&A cases were included as they had a pre-operative home oximetry with a MOS 1 (i.e. “normal/inconclusive”) available on record (Figure 1). Baseline demographic and clinical characteristics for patients operated at each hospital center are shown in Table 1. Of these 374 patients, 29 were excluded because of age under 3 years. Of the remaining 345, 90 were excluded due to home oximetry results that had been performed over 12 months prior to surgery. Of the remaining 255, 13 were excluded due to important comorbid conditions as outlined in the Methods, and 11 were excluded because, despite their MOS of 1, their treating surgeon deemed it necessary to undergo further testing prior to surgery (i.e. PSG) which was consistent with moderate-severe OSA and their oximetry was deemed a false negative.

**Table 1:** Baseline demographic and clinical characteristics between Montreal children’s hospital and Verdun hospital groups

**Variable, n(%)**	**MCH group (n=109)**	**Verdun group (n=122)**	**P value**
Age (mean ± SD)	5.63 ± 2.88	6.02 ± 2.72	0.289
Sex			
Male	62 (56.9%)	61 (50.0%)	0.295
Female	47 (43.1%)	61 (50.0%)	
Above 95^th^ percentile for weight-for-age	27 (24.8%)	21 (17.2%)	0.158
Associated medical condition (asthma n=19 and Turner’s Syndrome (n=1)	16 (14.7%)	4 (3.3%)	0.002

Of the 231 remaining eligible cases, 9 were tonsillectomies and 222 were T&A. The patients’ median age was 5 years and the mean length of surgery was 40.1 minutes (SD=15.0 minutes). There were 48 patients above the 95th percentile for their weight-for-age. Nineteen patients had asthma at the time of surgery, and one had Turner’s syndrome.

All characteristics at baseline were comparable between the MCH and the Verdun groups (Table 1) except for associated medical conditions that included asthma (19) and Turner’s Syndrome (1) (p = 0.002).

No patient in our study had a major respiratory complication. 226 out of the 231 (97.8%) eligible patients had no post-operative respiratory complications, and the remaining 5 had minor respiratory complications occurring within hours of surgery (Table 2). The minor respiratory complications were as follows: requiring supplemental oxygen (3) or humidity (1); requiring oral or nasal airway insertion (2); requiring bagging (1); requiring a jaw thrust manoeuvre (1); and admission to ward for observation because of witnessed respiratory compromise (1).

**Table 2:** Demographic data for patients with minor post-operative respiratory complications

**Patient**	**Age (year)**	**Sex**	**Associated respiratory medical condition**	**Surgery**	**Weight-for-age 95**^**th **^**percentile**	**Postoperative complications**	**Admitted to ward**	**Intervention on ward**
1	3	M	None	T&A	Under	Desat<90%, O_2_, nasal airway	Yes	7 hours O_2_ administration by nasal prongs
2	7	M	None	T&A	Under	Desat<90%, bagging	No	---
3	3	M	None	T&A	Under	Humidity	No	---
4	3	F	None	T&A	Under	O_2_	No	---
5	5	M	None	T&A	Under	Jaw thrust, oral airway, O_2_	No	---

Of the 5 patients with minor respiratory complications (Table 2), 4 were discharged home directly from the recovery room and 1 required admission for repeated oxygen desaturations when sleeping in the recovery room. He required supplemental oxygen and temporary insertion of a nasal airway. He was admitted to the ward floor in stable condition where he received supplemental oxygen by nasal prongs for 7 hours post-operatively. No adverse events occurred on the ward and he was discharged home the following day.

Three children were admitted for reasons unrelated to their post-operative respiratory status: one child was admitted for observation of suspected tonsillar bed bleed and the other 2 were admitted for pain control.

Two patients were admitted on the ward for reasons that could not be elicited upon reviewing their charts. Neither had any evidence of post-operative respiratory compromise that might explain reason for admission. Neither had any respiratory difficulty once admitted nor did they require supplemental oxygen.

Charts were also reviewed for evidence that patients had returned to hospital (i.e. ER or ENT clinic within the hospital) with respiratory complaints. No such patients were identified.

## Discussion

None of the patients had any major respiratory complications. Of the 5 patients who had minor respiratory complications, only 1 could not have been managed at an outpatient surgical center because he required specialized medical care outside of the immediate post-operative period. However, this patient was in stable condition throughout the post-operative course and could have been safely transferred to a facility with admitting beds if he had been operated as an outpatient.

The 5 remaining admitted patients (one for observation of minor bleed, 2 for pain control and 2 admitted for unknown reasons) were also stable post-operatively and could arguably have been managed at a peripheral center with the possibility of transfer for ward admission. With regards to the patient admitted for observation of a minor bleed, it should be noted that while bleeding is an important post-operative complication following T&A, it has been widely studied and is not considered a contraindication to same-day surgery in low-risk patients with short-term recovery room monitoring [20-22].

Furthermore, recent literature considers a general complication rate of 7.9% and an unplanned admission rate of 8.5% for children over the age of 3 undergoing T&A as acceptable for outpatient management [22]. Therefore, our rate of 2.16% (5 out of 231) and 2.16% (5 out of 231) for respiratory complications and ward admissions respectively in a carefully pre-selected subgroup is reasonable.

It must be noted that 11 patients were excluded from the analysis because, despite a MOS of 1, their treating surgeons chose to pursue further diagnostic testing (PSG) that demonstrated moderate-severe OSA. We chose to exclude these patients from our analysis for the following reasons: the limited sensitivity of overnight oximetry for detecting OSA is well recognized [23-25]. As such, professional judgment still has a strong role to play in the management of SDB where PSG is not available [6,19]. Therefore, it is reasonable to pursue further testing after a MOS of 1 if clinical suspicion remains high. However, when a MOS of 1 is paired with a low clinical suspicion in a carefully selected population, the risk of post-operative respiratory complications is very low and it is for these milder cases that our study offers the most valuable insight.

We noticed that none of the 5 patients with respiratory complications were obese. While obesity has been shown in previous studies to be a risk factor for minor respiratory complications post-T&A [26,27], a series of 26 morbidly obese patients by Shine et al. showed that the patients with respiratory complications post-T&A had an oxygen saturation nadir of <70% [27], which would correspond to a MOS 4 [18]. Thus, it is likely that obese patients who would have been susceptible to post-operative respiratory complications would likely have obtained a MOS greater than 1, and thus were excluded from our study. This further reinforces our findings showing the utility of the preoperative overnight oximetry.

Most of the 2334 patients we excluded for not having a MOS 1 did not have any oximetry study. Only a small portion of patients were excluded for moderate or severe OSA (MOS 3 or 4). Among the available oximetries, less than 20% of oximetries were of MOS 3 or 4. Due to the retrospective nature of this study, it was not possible to determine why not all children who underwent T&A had a pre-operative overnight oximetry test. It is known that some of the children underwent their oximetries privately but this information was not universally available. During the time period reviewed, not all surgeons requested oximetry study, out of individual preference. Once again, individual physician judgment likely played a strong role. A prospective study would address this particular issue more clearly.

The retrospective nature of this study also made it impossible to determine if medical interventions that were performed post-operatively were either appropriate or effective. Furthermore, chart review is not a very sensitive method for detecting minor interventions such as jaw thrust and may underestimate the number that were in fact performed. However, we can be confident of having identified all major outcomes of interest such as reintubation or transfer to the ICU that would preclude operating in peripheral hospital centers.

With regards to the discrepancy between the 2 groups of patients (from Verdun and from the MCH) in terms of associated medical conditions, both groups were from the same referral pool. Furthermore, all children were initially evaluated at the MCH and surgeries at both sites were performed by MCH surgeons.

One last important caveat is that all the surgeries in this study were performed by University hospital otorlaryngologists and in half the cases, within a University hospital. As a result, our findings may not be extrapolatable to community surgical centers.

As mentioned previously, it has been well documented that overnight oximetry has a low sensitivity for detecting OSA [23-25] whereas PSG has long been considered the gold standard for definitive diagnosis. However, PSG is a testing modality with important limitations because of its cost, long waiting times and lack of necessary equipment in many centers [6]. A recent survey suggests that only about 10% of pediatric oto-laryngologists obtain a preoperative PSG before tonsillectomy for SDB for these reasons [6].

Alternatively, overnight oximetry is inexpensive, easily implemented and widely available. Because of this, our group has been using overnight oximetry studies (without referral to more detailed PSG) to help predict the occurrence of post-operative respiratory complications in children with symptoms of OSA [18]. We believe our study supports the validity of this practice as long as the physician’s clinical suspicion for OSA remains low.

Of the 231 patients, only one or 0.43% (95% CI 0.08% to 2.41%) required ward admission for respiratory support. This patient was stable throughout the post-operative period and could theoretically have been transferred to an admitting facility if his surgery had been performed as an outpatient. The findings suggest that the MOS can be used as screening tool to determine which patients with SDB (in whom physicians have a low index of suspicion for OSA) can be safely sent to peripheral hospitals or outpatient surgical centers for T&A as long as these are within reasonable distance of a facility where hospitalization may be readily facilitated. These patients must be 3 years and older, have a MOS of 1 performed less than a year before surgery, and must not have important severe comorbidities.

## Conclusion

For children who do not fit very specific criteria, post-operative protocol is still very much at the discretion of the treating surgeon and there is currently a significant amount of variation between centers and individual physicians [6,9]. It is our hope that the findings of this study will arm physicians with a practical and accessible tool to triage patients who could be operated on at peripheral hospitals or outpatient surgical centers. This would then significantly relieve the caseload from tertiary centers. Overall, this will reduce surgical wait times for children with SDB with potentially important impacts on long-term cognitive function.

## Abbreviations

SDB: Sleep-disordered breathing; OSA: Obstructive sleep apnea; PSG: Polysomnography; T&A: Adenotonsillectomy; MOS: McGill oximetry score; MCH: Montreal children’s hospital; BPD: Bronchopulmonary dysplasia; ICU: Intensive care unit; URTI: Upper respiratory tract infection; CPAP: Continuous positive airway pressure; PACU: Post-anesthesia care unit.

## Competing interests

This study had no financial sponsorship and we have no potential conflicts of interest to declare.

## Authors’ contributions

SJ Daniel contributed to conception and design of this study, revised the article and approved this final version for submission. CE Lefebvre contributed to acquisition and interpretation of data, drafted the article and approved this final version for submission. TTN Lee contributed to acquisition and interpretation of data, drafted the article and approved this final version for submission. NE Gans contributed to acquisition and interpretation of data, and approved this final version for submission.
